# Comment on Islam et al. Helminth Parasites among Rodents in the Middle East Countries: A Systematic Review and Meta-Analysis. *Animals* 2020, *10*, 2342

**DOI:** 10.3390/ani13223443

**Published:** 2023-11-08

**Authors:** Yazdan Hamzavi, Arezoo Bozorgomid, Mosayeb Rostamian

**Affiliations:** Infectious Diseases Research Center, Health Institute, Kermanshah University of Medical Sciences, Kermanshah 6714415333, Iran; yhamzavi@kums.ac.ir (Y.H.); arezoobozorgomid@yahoo.com (A.B.)

I read with great interest the article by Islam et al. entitled “Helminth parasites among rodents in the Middle East countries: a systematic review and meta-analysis” published in *Animals* in December 2020 [1]. The authors of the article conducted a systematic review and meta-analysis of studies that investigated helminthic parasites in rodents in the Middle East region. They reported the overall prevalence of cestodes, nematodes, and trematodes as 24.88%, 32.71%, and 10.17%, respectively. The article provides important information on the prevalence of helminthic parasites in rodents in the Middle East, which can have implications for human health as some of these parasites can be zoonotic and may cause diseases in humans. The findings of the study can help inform public health policies and interventions aimed at controlling and preventing the spread of these parasites. However, the results obtained from the included studies in the systematic review (Figures 3 to 5 and related tables) are reported incorrectly, as described below.

The prevalence of rodent cestode infection is defined as “the number of infected rodents with at least one cestode/the total number of rodents × 100”. It does not matter if one genus or several different genera of cestode is/are isolated from a rodent, in both cases, we report the rodent as infected with cestodes. Only a small number of included studies in the Islam et al. meta-analysis have directly reported the overall prevalence of infection with different classes of rodent parasites (cestodes, trematodes, or nematodes). Instead, many of them have only reported the prevalence of rodent infection with separate genera and species (and not overall prevalence with classes) of parasites. Therefore, the prevalence of infection with parasite classes cannot be calculated based on the results of these included articles.

To clarify the issue, consider the study of Nateghpour et al. [2], which is one of the included studies in the meta-analysis. In this study, researchers examined 100 rodents and reported that eight rodents were infected with *Hymenolepis nana feraterna*, and eleven rodents were infected with *Hymenolepis diminuta*. We cannot sum the frequency of rodent infection with *H. nana* (*n* = 8) and *H. diminuta* (*n* = 11) and report it as the prevalence of cestode infection in this study (*n* = 19) because a rodent may be infected with both *H. nana* and *H. diminuta* cestodes at the same time. However, in Figure 3, Islam et al. wrongly reported a rate of cestode infection of 19% for the study of Nateghpour et al. As mentioned above, this only applies if it is acknowledged that only one parasite was isolated from each rodent (which, according to the evidence of Nateghpour et al.’s article, does not seem to be the case).

Several similar errors that occurred in the data of other articles that were included in the results of the meta-analysis can be seen in the meta-analysis (Figures 3 to 5 and related tables). Such errors cause inaccuracies in all estimates related to the prevalence of parasites in rodents and therefore lead to inaccuracies in the entire meta-analysis results.
